# Trends in Israel's Medical Administration subspecialty, 1987–2022

**DOI:** 10.1186/s13584-025-00666-8

**Published:** 2025-01-13

**Authors:** Yoel Angel, Hadar Goldshtein, Nevo Barel, Gil Fire, Michael Halberthal, Adi Niv-Yagoda

**Affiliations:** 1 Tel Aviv Sourasky Medical Center, Tel Aviv, Israel; 2 School of Medicine, Faculty of Medical and Health Sciences and the Coller School of Management, Tel Aviv University, Tel Aviv, Israel; 3 Ben Gurion University, Beer Sheva, Israel; 4 Rambam Healthcare Campus, Haifa, Israel; 5 The Ruth & Bruce Rappaport Faculty of Medicine, Technion-Israel Institute of Technology, Haifa, Israel; 6 School of Health Systems Management, Netanya Academic College, Netanya, Israel

**Keywords:** Israel, Healthcare administration, Physician leadership, Hospital governance, Health policy

## Abstract

**Background:**

Israel is unique in offering a formal subspecialty in Medical Administration and mandating it for physicians applying for senior roles. Data on the prevalence and characteristics of these specialists are limited.

**Methods:**

The national registry of licensed physicians was used to identify all living physicians who completed the Medical Administration subspecialty by December 31, 2022. Data on year of medical licensing, city of residence, and list of additional recognized specialties along with their respective date of completion were extracted. Websites of key public health organizations were sampled to identify qualifications of persons in senior leadership positions.

**Results:**

Since 1987, 277 physicians have completed the Medical Administration subspecialty, with a significant increase in annual certifications from 4.5 in 2015 (interquartile range [IQR] 4–6) to 13 (IQR 10.5–15) in 2022 (*p* < 0.001). Specialists completed the subspecialty a median of 18 years (IQR 13–21) post-licensing, with 269 physicians (97.1%) holding additional specialties, primarily in Internal Medicine, Pediatrics, Family Medicine, or Public Health. Compared to the general physician population, some base specialties like Public Health are over-represented while others, like Anesthesiology, are under-represented. Only 40 (14.4%) specialists reside outside major metropolitan areas. Nineteen (61.3%) general hospital CEOs, 2 (20%) psychiatric hospital CEOs, 13 (35.1%) Ministry of Health and 4 (7.8%) Sick Fund executives are specialists in Medical Administration (*p* < 0.005).

**Conclusions:**

The steady growth in the number of specialists in Medical Administration demonstrates the sustainability and scalability of this model, which may serve as a template for other healthcare systems. However, the limited representation of these specialists in senior roles of some organizations, and their concentration within certain specialties and regions, indicates areas for policy attention to enhance leadership diversity and reduce healthcare disparities.

**Supplementary Information:**

The online version contains supplementary material available at 10.1186/s13584-025-00666-8.

## Background

Traditionally, senior positions in the Israeli healthcare system have been filled by physicians, including senior management roles in Israeli hospitals, Sick Funds and Ministry of Health (MoH). This may be traced back to the early days of the state of Israel, when the originally British law stated that “Every hospital shall be under the supervision of a licensed physician” [1]. All government-owned hospitals in Israel are run by physicians, a policy which was ratified by the Supreme Court in 2017 [2]; In the Sick Funds, Ministry of Health and non-government hospitals, senior management positions are filled by physicians and non-physicians in a more balanced mix.

In the 1980’s, it became evident that to successfully navigate the growing complexity of healthcare administration, physicians in senior positions need formal management training. Being one of the first countries to identify this lacune, Israel established formalized training in leadership and management for physicians: In the early 1980s, the first post-graduate training in Medical Administration was established in the University of Haifa; and In 1987, the Israeli MoH together with the Scientific Council of the Israeli Medical Association established a recognized subspecialty in Medical Administration, the first of its kind in the world. Nowadays, possession of this subspecialty is one of the prerequisites to apply for leadership roles in the Israeli healthcare system [3, 4]. The first to be acknowledged as specialists, those who were defined as “founders of the field”, were physicians who filled senior leadership roles in the Israeli healthcare system at that time. From 1992 onwards, physicians were required to have at least one other medical specialty to apply for a residency in Medical Administration. The training process has not changed significantly since then, and it entails, like most other clinical subspecialities, a two-year fellowship program in the senior management of two different healthcare organization in Israel (Hospitals, Sick Funds or the Ministry of Health) under the supervision of a certified specialist in Medical Administration; an advanced degree in healthcare administration such as a Master's degree in public health (MPH), health administration (MHA) or business administration (MBA); and successful passage of an oral and written examination^5^. While the initial requirements from residents were vague, the residency syllabus and final exams were refined during 1990–2000 to better match the competencies expected from specialists. Today, the stated objectives of the residency are to provide residents with the skills, knowledge and behavior required to fulfill managerial roles in the healthcare system, across multiple dimensions such as interpersonal leadership, data analysis, decision making and more [5].

There is no published data to date on the number of physicians in Israel with a recognized subspecialty in Medical Administration, their characteristics, and the trends in application to this subspecialty since its establishment. This study aims to shed light on these data and review their implications on the field of Medical Administration in Israel.

## Methods

This was a retrospective study evaluating the trends in number and characteristics of physicians with a subspecialty in Medical Administration.

The publicly accessible national registry of licensed physicians was accessed through the Israeli Ministry of Health (MoH) website [6] during February 2023. Data on all living physicians with a recognized subspecialty in Medical Administration listed in the registry by December 31st 2022 was extracted. Data obtained for each physician included full name, year of medical licensing, city of residence, and list of recognized specialties along with their respective date of listing in the registry. Further manipulations were performed on the data, such as classification of city of residence by geographical district or calculation of time between physician’s different professional milestones. Descriptive statistics were extracted from the data. Physicians who previously obtained this subspecialty but were not alive during the conduction of the survey do not appear in the MoH’s database and therefore were not included in the study.

To explore the representation of specialists in Medical Administration among senior leadership positions, the websites of all public general hospitals (n = 31), public psychiatric hospitals (n = 10), the four Sick Funds and the MoH were sampled during October 2024 to identify persons in key leadership positions, which were defined as hospital CEOs; Sick Funds` CEOs, deputy directors and district managers; and the CEO, Deputy Directors, Division Managers and District Managers of the MoH. For the sake of consistency and to avoid selection bias, all deputy directors, division and district managers were included, even when they oversee clearly non-clinical fields (e.g. Information Technology or Finance). Names in this list were matched against the list of specialists in Medical Administration updated as of October 2024.

To draft the background for this paper, a search for pertinent documents was made in the websites of the Israeli Ministry of Health and the Israeli Medical Association, and representatives of these organizations were contacted to search for additional documents in internal databases. As very few documents from the relevant period were available, unstructured interviews were performed by one or more of the authors with current and former heads of the Israeli Association of Medical Administration as well as prominent figures in the Israeli healthcare system that were involved in the inception or foundation of the subspecialty in Medical Administration. The list of persons interviewed appears in the “acknowledgements” section below.

### Statistical analysis

Continuous non–normally distributed variables are expressed as median (interquartile range). Categorical variables are expressed as number (percentage) within each group. Groups were compared the Mann–Whitney U test for continuous variables and the χ^2^ test for categorical variables. All reported tests were 2-sided and a *p* value of less than 0.05 was considered significant. All statistical analyses were performed using R software version 4.0.3 (R Foundation for Statistical Computing) or SPSS version 28 (IBM Corporation, Armonk, NY).

## Results

A total of 278 physicians were listed in the MoH's database as specialists in Medical Administration by December 31st, 2022. One physician was listed as accredited in Medical Administration in 1981 (6 years prior to the foundation of this subspecialty), and consequently was considered an outlier and removed from all further analyses.

### Temporal distribution of new licenses

**Fig. 1:**
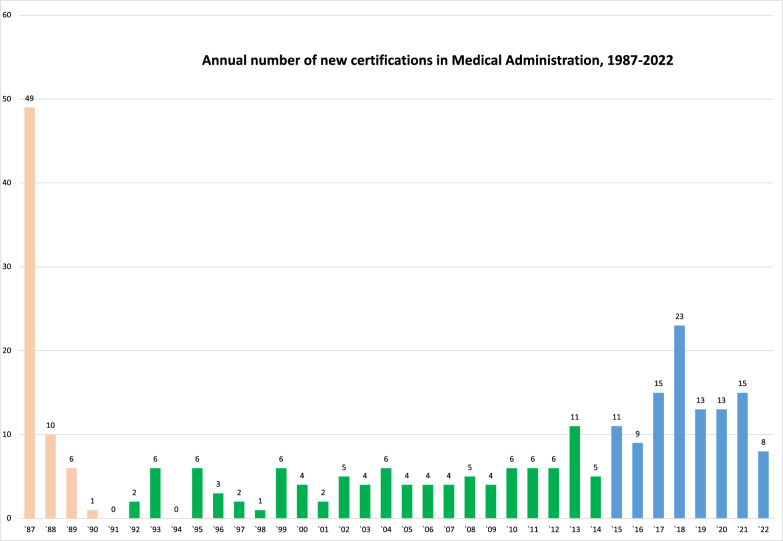
Annual number of newly registered specialists in Medical Administration. The pink bars represent specialists listed during period A, green bars represent period B, and blue bars period C

Between the years 1987–2022, three distinct periods could be identified considering the annual number of new specialists in Medical Administration (Fig. 1). Period A (1987–1991), which included the “founders of the field”. Period B (1992–2014), with a median of 4.5 (IQR 4–6) new specialists each year, and period C (2015–2022) with a median of 13 new specialists each year (IQR 10.5–15), significantly more than period B (*p* < 0.001).

### Additional specialties

**Fig. 2:**
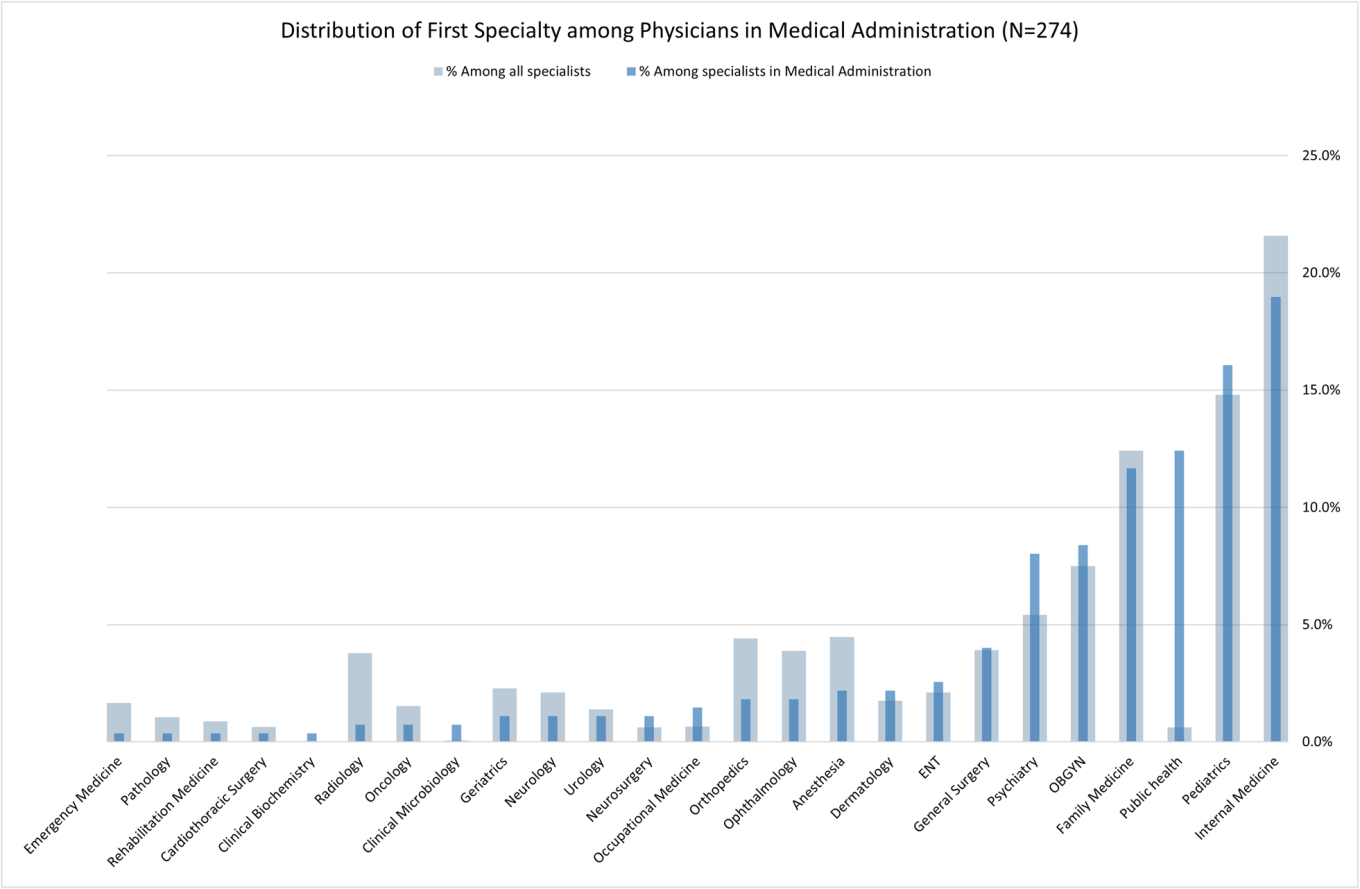
Blue: Proportion of additional base specialties held by physicians with a specialty in Medical Administration (N = 274). Grey: proportion of specialties in each field among all specialist physicians in Israel under the age of 67, according to 2021 Ministry of Health data [7]. See also Figure S1 in the Supplementary Appendix

Two hundred and sixty-nine (97.1%) physicians had at least one other specialty. Among these, 215 (77.6%) had one additional specialty; 48 (17.3%) had 2 additional specialties or subspecialties and 6 (2.1%) had 3 additional specialties or subspecialties. Approximately 60% of physicians were specialists in one of four specialties—Internal Medicine, Pediatrics, Public Health, and Family Medicine. Compared to proportions among all 17,369 specialist physicians under the age of 67 included in the 2021 Ministry of Health report [7], the proportion of physicians with a specialty in Public Health was greater (12.4% versus 0.6%), while the proportions for the other 3 specialties were similar (Fig. 2 and Fig. S1 in the Supplementary Appendix).

### Timing of subspecialty in medical administration

**Fig. 3:**
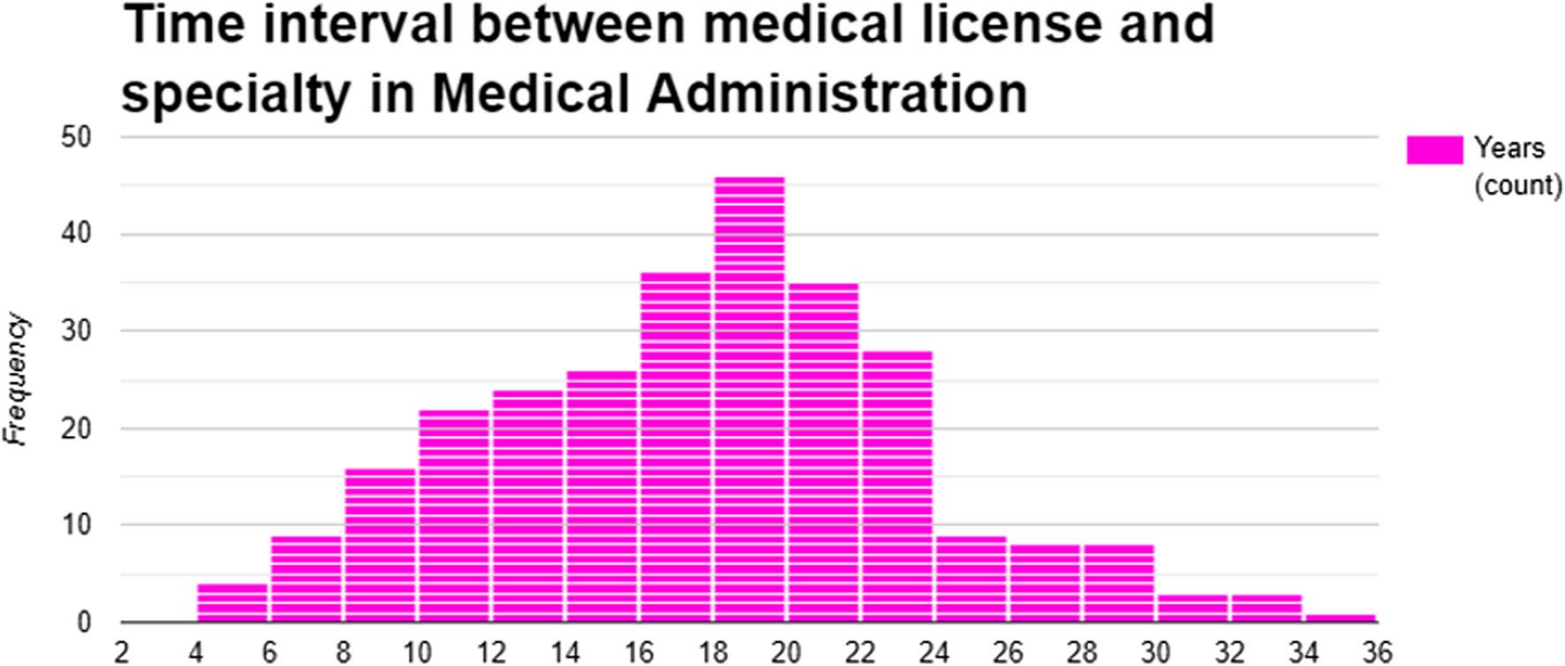
A histogram showing time between acquisition of medical license and time of specialty in Medical Administration (N = 277). See also Fig. S2 in the Supplementary Appendix

The median time between obtaining a medical license and completion of a subspecialty in Medical Administration was 18 years (IQR 13–21 years; Fig. 3). This interval was significantly shorter in period B, 17 (IQR 13–19) years, compared to period C—19 (IQR 14–23) years, *p* = 0.006 (Fig. S2).

**Fig. 4:**
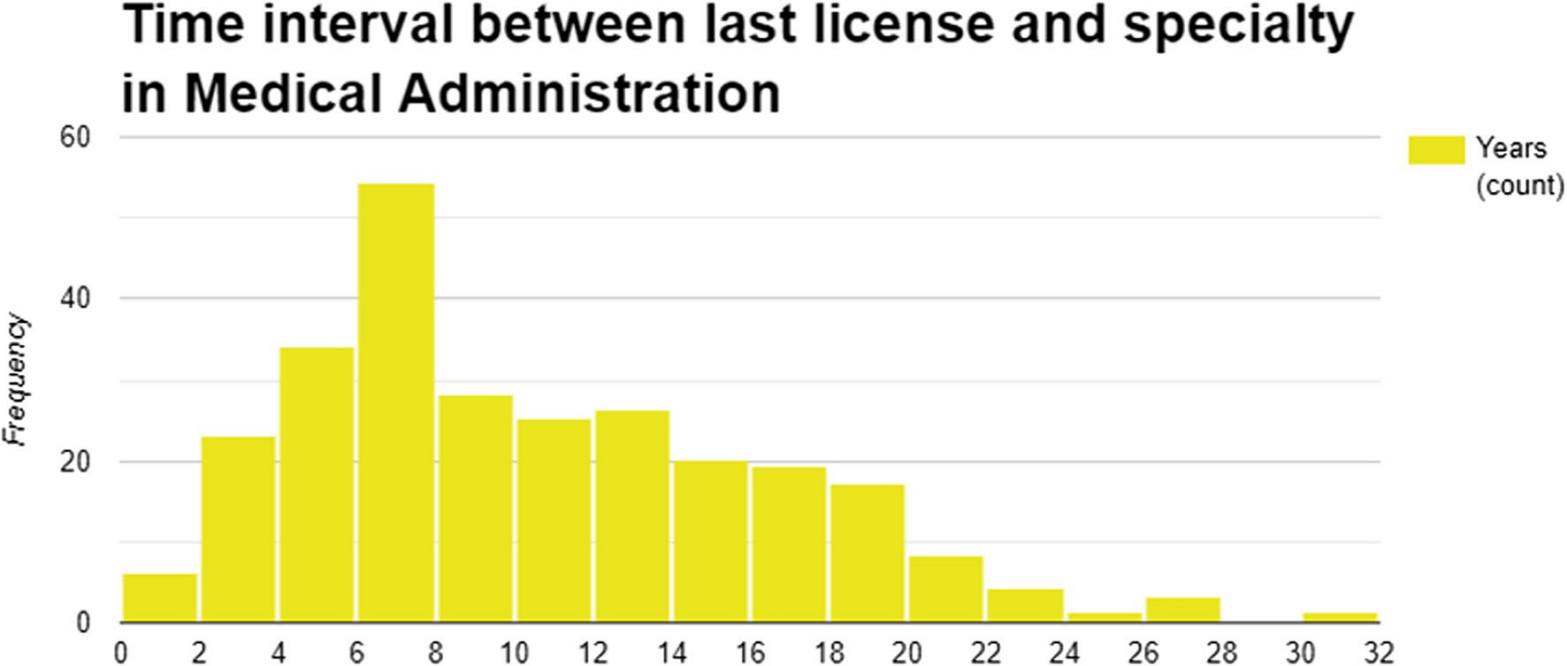
A histogram showing time between completion of last base specialty/subspecialty and time of completion of specialty in Medical Administration (N = 277). See also Figure S3 in the Supplementary Appendix

The median time between completion of base specialty and completion of a subspecialty in Medical Administration was 9 years (IQR 6–14 years; Fig. 4). The median interval in period C was 10 (IQR 6–16) years compared to 7.5 (IQR 6–12) years in period B and 8 (IQR 4–14) years in period A, however this trend did not reach statistical significance (*p* = 0.052; Figure S3).

Eighteen physicians (6.5%) obtained another specialty after being certified in Medical Administration, but only 2 of these were not “founders of the field”.

### Geographical distribution

170 (61.4%) of physicians were listed to reside in the center of Israel; 38 (13.7%) were listed in the Haifa region and 29 (10.5%) listed in the Jerusalem region; the remaining 40 (14.4%) physicians were listed to reside in northern or southern Israel.

### Representation of specialists in medical administration in senior leadership roles

Of 31 Public General Hospitals, 28 (90.3%) of CEOs are physicians, and 19 (61.3%) are specialists in Medical Administration. Of 10 Psychiatric Hospitals, 10 (100%) are physicians and 2 (20%) are specialists in Medical Administration. None of the four sick funds are managed by physicians, and among the 51 Sick Fund executives sampled, 11 (21.6%) are physicians and 4 (7.8%) are specialists in Medical Administration. Of 37 executives in the MoH, 21 (56.7%) are physicians, and 13 (35.1%) are specialists in Medical Administration. The proportion of physicians and of specialists in Medical Administration significantly differed between the 5 organization types (*p* < 0.001. Table S1 in the Supplementary Appendix).

## Discussion

This study describes the inception of the Medical Administration subspecialty in Israel and the characteristics of physicians who pursued this path. After the qualification of the initial cohort of "founders of the field" (1987–1991), the annual number of new specialists remained relatively constant at a median of 4.5 until approximately 2015, when it increased sharply to a median of 13 new specialists annually. This increase may have resulted from the MoH regulation, issued in 2006–2008 and effective 2013, mandating Medical Administration subspecialty certification for senior leadership roles [3, 4] and the establishment of the Inbar fellowship program for leaders in the public health sector [8] in 2015, which initially included such certification and significantly raised awareness to it.

To our knowledge, Israel is the only country that offers a formal residency in Medical Administration, and the only country that requires management training to serve in senior leadership positions. Healthcare systems in other countries take different approaches to leadership development: In the United States, physicians in leadership positions often complete business training such as MBA or MHA degrees, and may also pursue voluntary board certification in healthcare management [9]. Still, none of these qualifications are mandatory, and the hands-on training component, if included, differs across programs. Similarly, the UK's NHS Leadership Academy and the Faculty of Medical Leadership & Management provide structured programs of varying duration, though these are not formalized as medical subspecialties and are also offered to non-physician healthcare professionals [10]. The situation in other European countries is generally comparable.

By offering this training in the form of a two-year residency, which includes uniform examinations and guided hands-on experience, the Israeli model offers standardized, supervised and scalable training, while also strengthening the health ecosystem by promoting networking among its emerging leaders.

Israel's public healthcare system is known for its good health outcomes and efficient spending compared to other developed countries [11, 12]. Although the direct impact of Medical Administration training on health outcomes is unclear, evidence from other countries suggests that physician-led healthcare organizations generally perform better in areas like patient satisfaction, care quality, and spending [13–17]. Possible contributing factors include better understanding of the medical field, a more patient-centric or ethics-based approach to decision-making, and greater trust from medical professional employees [18]. This aligns with the Theory of Expert Leadership by Goodall[19], which suggests that organizations led by experts, such as clinically trained physicians, perform better.

Despite the 2013 MoH regulation [3, 4], only 61% of general hospital managers and 7.8% of Sick Fund executives are specialists in Medical Administration, albeit our sample did not include medical managers who report to non-physician CEOs. Many other specialists likely hold middle-management positions and may still have a positive impact on organizational performance—though this requires empirical validation. Nevertheless, given the advantages of physician leadership, integration of specialists in Medical Administration into more senior executive roles within Sick Funds and non-government hospitals should be prioritized. Moreover, with the increase in total number of specialists in Medical Administration, and particularly psychiatrists (Fig. 2), the MoH should also reconsider the exclusion of psychiatric hospitals from the 2013 regulation, which was due to a lack of suitable candidates at that time.

One of the main challenges for the Israeli healthcare system is reducing disparities in access and quality across the country. 61.4% of specialists in Medical Administration come from the center of Israel, while only 40% of the population and 47.5% of physicians reside in this area [7, 20]. Increasing representation of healthcare executives from underserved regions is an important area for improvement. Furthermore, over 50% of specialists come from fields like Internal Medicine, Pediatrics, Family Medicine, and Public Health. Other fields are under-represented: Anesthesiologists, for example, comprise 4.5% of all specialists in Israel, and the field is often cited as suffering from a serious workforce shortage [21]. Yet, only 2.2% of specialists in Medical Administration are anesthesiologists. This uneven distribution, both geographically and by specialty, could exacerbate existing disparities, and addressing this imbalance merits consideration.

Pursuing a subspecialty in Medical Administration seems to occur late in a physician's career, unlike other subspecialties typically obtained soon after completing base specialty training. This suggests a career shift for accomplished clinicians. Motivations may include a desire for broader influence, an alternative promotion path, or burnout in clinical roles—a significant concern in the Israeli healthcare system[22]. Since 2015, more physicians have pursued this subspecialty later in their careers, indicating a potential change in motivations which needs to be characterized.

### Areas for future research

There are several important unanswered questions that future studies should address. First, to what extent do healthcare organizations led by physicians with management training, particularly specialists in Medical Administration, outperform those led by physicians without such training? Such a comparison should cover multiple facets of healthcare delivery including (but not limited to) clinical, financial and patient and staff satisfaction metrics. Second, does the theory of expert leadership apply only to physician leadership, or do organizations led by other healthcare professionals (e.g., nurses) perform similarly? Lastly, further research is needed to understand clinicians' motivations for pursuing a subspecialty in Medical Administration and to assess how this subspecialty impacts their skillset and career trajectory.

### Strengths and limitations

The key strength of this study is the systematic complete census of all living physicians with a subspecialty in Medical Administration in Israel and of all senior positions in the Sick Funds, MoH and General and Psychiatric Hospitals. Nonetheless, some limitations should be noted: Firstly, the data available through the MoH website lacks many basic demographic details such as age and gender, limiting interpretation of the data. Secondly, this study did not use a control group of physicians of other specialties, as using a similar manual method was impractical and we did not have direct access to the MoH dataset. Lastly, deceased specialists were excluded, as their licenses are no longer listed in the public registry.

## Conclusions

The steady growth in the number of specialists in Medical Administration, particularly in recent years, demonstrates that this subspecialty is a sustainable and scalable model for physician management training, potentially replicable in other healthcare systems. However, the underrepresentation of these specialists in senior roles in some organizations, and the unequal distribution across specialties and regions require attention from the MoH, as they may hinder efforts to improve performance and reduce healthcare disparities. Further research into the motivations and career paths of specialists in Medical Administration could provide valuable insights for improving healthcare leadership and management.

## Supplementary Information

Additional file 1.

## Data Availability

The data supporting this study is publicly available from the Israeli Ministry of Health’s National Registry of Licensed Physicians (in Hebrew), at https://practitioners.health.gov.il/Practitioners.
