# Supplementary material for: Trends in Israel's Medical Administration subspecialty, 1987–2022
Source: Isr J Health Policy Res. 2025 Jan 13;14:3. doi: 10.1186/s13584-025-00666-8 (PMC11730815; doi:10.1186/s13584-025-00666-8)
Supplement: Supplementary file 1 — Additional file 1. [file 13584_2025_666_MOESM1_ESM.pdf]

Trends in Israel's Medical Administration Subspecialty, 1987-2022

Yoel Angel, MD, MBA; Hadar Goldshtein, MA; Nevo Barel, MD, MHA; Gil Fire, MD, MBA; Michael Halberthal, MD, MHA; and Adi Niv-Yagoda, PhD, LL.M, LL.B

Corresponding Author:

Dr. Yoel Angel
yangel.md@gmail.com
+972-3-6947842

SUPPLEMENTARY APPENDIX

SUPPLEMENTARY FIGURES

Figure S1: Distribution of second specialties among physicians with a specialty in Medical Administration. Narrow bars: A histogram listing additional sub-specialties held by specialists in Medical Administration (N=45). Wide bars: proportion of specialties in each field among all specialists with 2 or more specialties under the age of 67 in Israel, according to 2021 Ministry of Health data[1] (N=4,068).

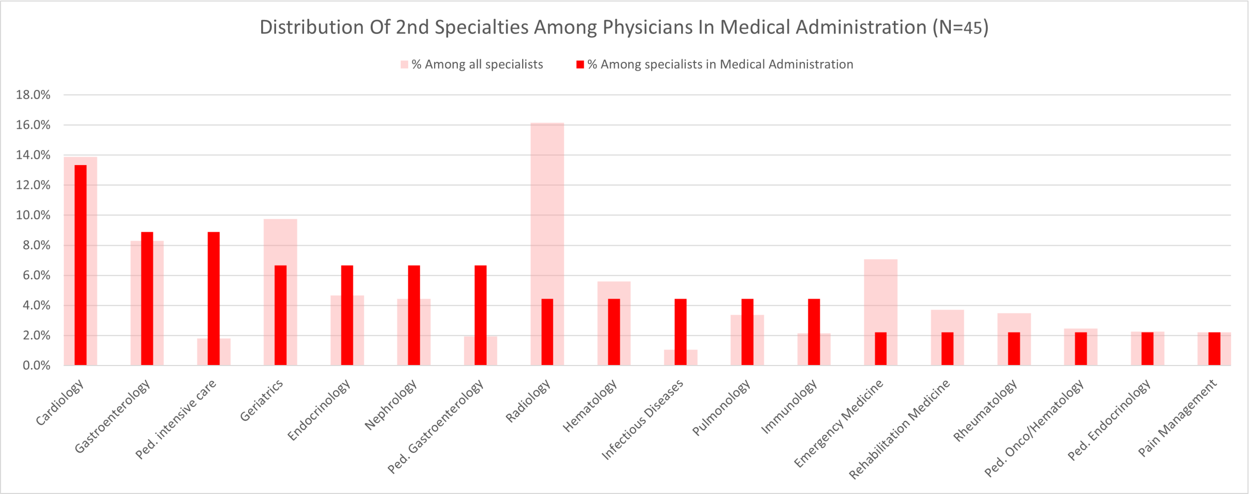

[1] the Israeli Ministry of Health. (2022). *Workforce in Health Professions in Israel, 2021 (Hebrew)*.

Figure S2: A panel of histograms showing time between acquisition of medical license and time of specialty in Medical Administration (N=277). Figure S2-A,B and C refer to periods A (1987-1991), B (1992-2014) and C (2015-2022), respectively.

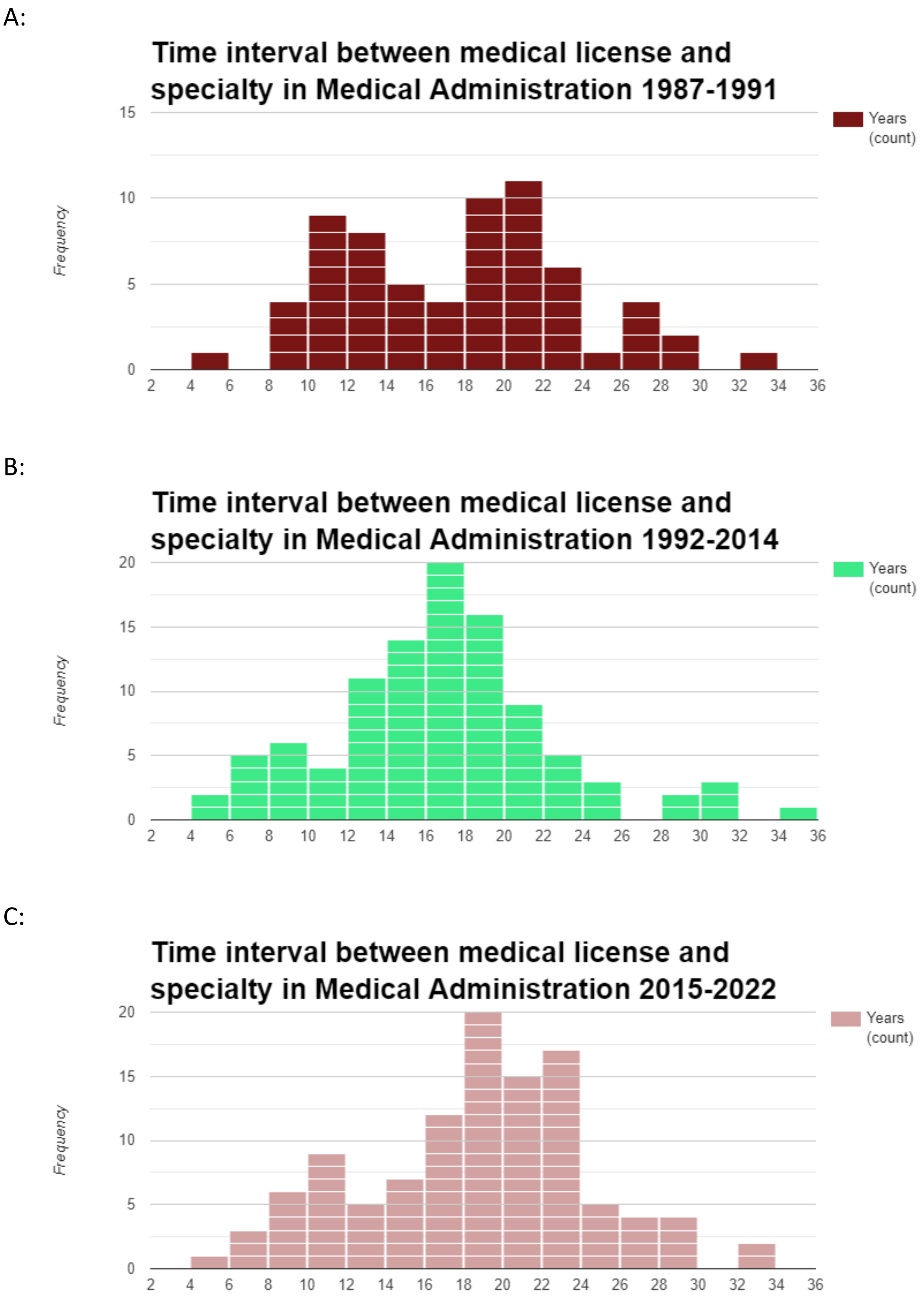

Figure S3: A panel of histograms showing time between completion of last specialty and time of completion specialty in Medical Administration (N=268). Figure S2-A,B and C refer to periods A (1987-1991), B (1992-2014) and C (2015-2022), respectively.

A:

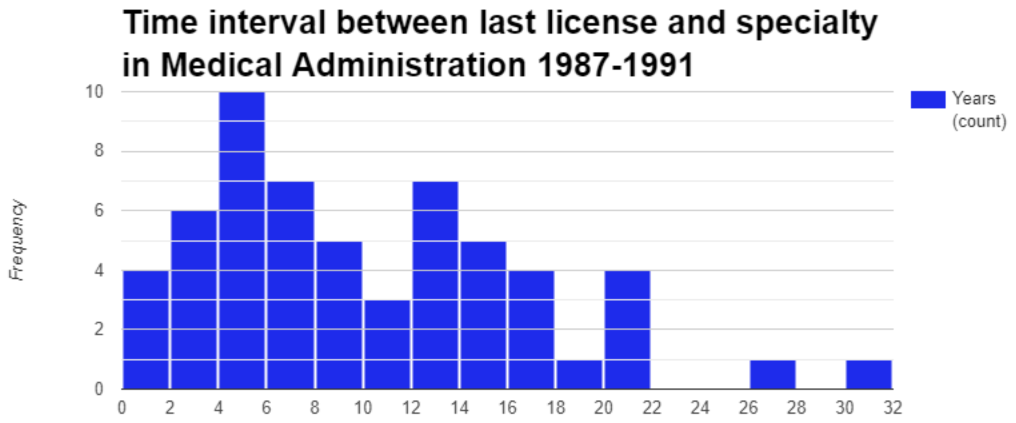

B:

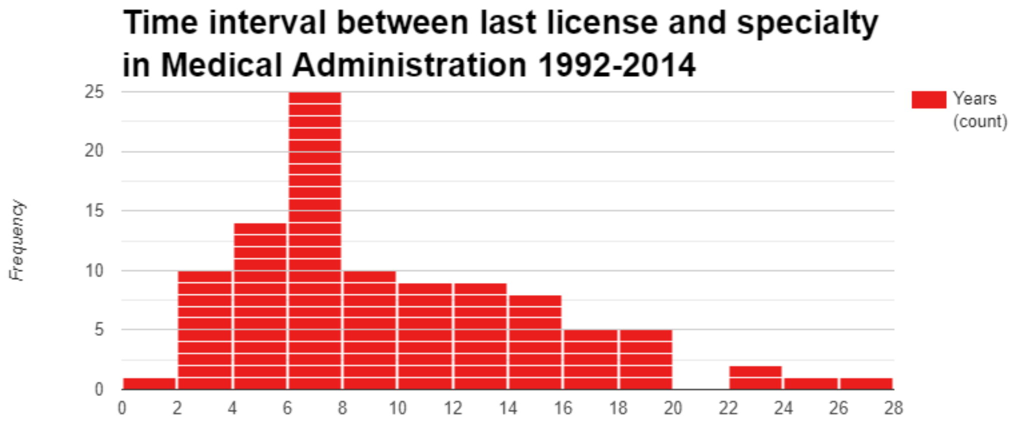

C:

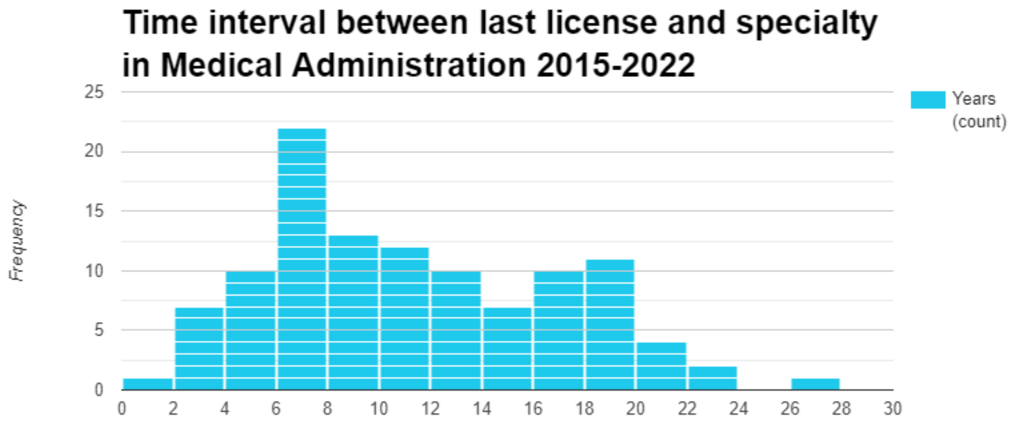

Table S1: Executives in General Hospitals, Publicly-owned Mental Health Institutions, Sick Funds and the Ministry of Health as of October 2024 and their specialty status (N=130).

Identity of position holders was extracted from each organization's publicly accessible website. Status of specialty in Medical Administration was validated against the Ministry of Health's online Specialist Registry. All data is updated to October 2024.

No.	Organization type	Organization	Position	Physician?	Specialist In Medical Administration?
1.	General Hospital	Assuta Ashdod Medical Center	CEO	Yes	Yes
2.	General Hospital	Barzilai Medical Center	CEO	Yes	No
3.	General Hospital	Beilinson Medical Center	CEO	Yes	No
4.	General Hospital	Bnai Zion Medical Center	CEO	Yes	Yes
5.	General Hospital	Carmel Medical Center	CEO	Yes	Yes
6.	General Hospital	Emek Medical Center	CEO	Yes	Yes
7.	General Hospital	French Hospital	CEO	Yes	No
8.	General Hospital	Hadassah Ein Kerem Medical Center	CEO	Yes	Yes
9.	General Hospital	Hadassah Medical Center	CEO	Yes	Yes
10.	General Hospital	Hadassah Mount Scoupus Medical Center	CEO	Yes	Yes
11.	General Hospital	Hasharon Medical Center	CEO	Yes	Yes
12.	General Hospital	Hillel Yaffe Medical Center	CEO	Yes	Yes
13.	General Hospital	Holy Family (Italian) Hospital	CEO	No	No
14.	General Hospital	Kaplan Medical Center	CEO	Yes	Yes
15.	General Hospital	Laniado Hospital	CEO	No	No

No.	Organization type	Organization	Position	Physician?	Specialist In Medical Administration?
16.	General Hospital	Mayanei Hayeshua Medical Center	CEO	No	No
17.	General Hospital	Meir Medical Center	CEO	Yes	Yes
18.	General Hospital	Nazareth Hospital (Emms)	CEO	Yes	No
19.	General Hospital	Rabin Medical Center	CEO	Yes	No
20.	General Hospital	Rambam Health Care Campus	CEO	Yes	Yes
21.	General Hospital	Shaare Zedek Medical Center	CEO	Yes	No
22.	General Hospital	Shamir - Assaf Harofeh Medical Center	CEO	Yes	Yes
23.	General Hospital	Sheba Tel Hashomer Medical Center	CEO	Yes	Yes
24.	General Hospital	Shneider Medical Center	CEO	Yes	Yes
25.	General Hospital	Soroka Medical Center	CEO	Yes	No
26.	General Hospital	Sourasky Medical Center (Ichilov)	CEO	Yes	Yes
27.	General Hospital	Tzafon Medical Center	CEO	Yes	Yes
28.	General Hospital	Western Galilee Medical Center	CEO	Yes	No
29.	General Hospital	Wolfson Medical Center	CEO	Yes	Yes
30.	General Hospital	Yoseftal Hospital	CEO	Yes	No
31.	General Hospital	Ziv Medical Center	CEO	Yes	Yes
32.	Psychiatric Hospital	Maale Hacarmel	CEO	Yes	Yes

No.	Organization type	Organization	Position	Physician?	Specialist In Medical Administration?
33.	Psychiatric Hospital	Mazor	CEO	Yes	No
34.	Psychiatric Hospital	Shaar Menashe	CEO	Yes	Yes
35.	Psychiatric Hospital	Lev Hasharon	CEO	Yes	No
36.	Psychiatric Hospital	Shalvata	CEO	Yes	No
37.	Psychiatric Hospital	Geha	CEO	Yes	No
38.	Psychiatric Hospital	Abarbanel	CEO	Yes	No
39.	Psychiatric Hospital	Beer Yakov	CEO	Yes	No
40.	Psychiatric Hospital	The Jerusalem mental health center	CEO	Yes	No
41.	Psychiatric Hospital	Beer Sheva mental health center	CEO	Yes	No
42.	Ministry of Health	Ministry Of Health	CEO	No	No
43.	Ministry of Health	Ministry Of Health	Deputy director general	Yes	Yes
44.	Ministry of Health	Ministry Of Health	Deputy Director General - Budgeting, Planning and Pricing	No	No
45.	Ministry of Health	Ministry Of Health	Deputy Director General of HMOs in the Ministry of Health	No	No
46.	Ministry of Health	Ministry Of Health	Director of Medical Technology and Infrastructure Administration	Yes	Yes
47.	Ministry of Health	Ministry Of Health	Director of Nursing Division	No	No
48.	Ministry of Health	Ministry Of Health	Director of Public Health	Yes	Yes
49.	Ministry of Health	Ministry Of Health	Director of the Nutrition Division	No	No

No.	Organization type	Organization	Position	Physician?	Specialist In Medical Administration?
50.	Ministry of Health	Ministry Of Health	Director of the Policy Planning Department	No	No
51.	Ministry of Health	Ministry Of Health	District Physician of the Central District	Yes	Yes
52.	Ministry of Health	Ministry Of Health	District Physician of the Haifa District	Yes	Yes
53.	Ministry of Health	Ministry Of Health	District Physician of the Jerusalem District	Yes	No
54.	Ministry of Health	Ministry Of Health	District Physician of the Northern District	Yes	No
55.	Ministry of Health	Ministry Of Health	District Physician of the southern Ashkelon District	Yes	Yes
56.	Ministry of Health	Ministry Of Health	District Physician of the southern Beer Sheva District	Yes	No
57.	Ministry of Health	Ministry Of Health	District Physician of the Tel Aviv District	Yes	No
58.	Ministry of Health	Ministry Of Health	Epidemiology Division Manager	Yes	Yes
59.	Ministry of Health	Ministry Of Health	Head of Community Medicine Division	Yes	Yes
60.	Ministry of Health	Ministry Of Health	Head of Department of Dental Health	No	No
61.	Ministry of Health	Ministry Of Health	head of digital health initiative	No	No
62.	Ministry of Health	Ministry Of Health	Head of Directorate of Government Medical Centers	Yes	Yes
63.	Ministry of Health	Ministry Of Health	Head of Division for Assessment of Technology in the Health Basket	No	No
64.	Ministry of Health	Ministry Of Health	Head of Division of General Medicine	Yes	Yes
65.	Ministry of Health	Ministry Of Health	Head of Division of Medical Technology Policy	No	No
66.	Ministry of Health	Ministry Of Health	Head of information division	No	No

No.	Organization type	Organization	Position	Physician?	Specialist In Medical Administration?
67.	Ministry of Health	Ministry Of Health	Head of the Division of Digital Technology and Data	No	No
68.	Ministry of Health	Ministry Of Health	Head of the General Medicine Division	Yes	Yes
69.	Ministry of Health	Ministry Of Health	Head of the Geriatrics Division	Yes	Yes
70.	Ministry of Health	Ministry Of Health	Head of the Medical Professions Licensing Division	Yes	No
71.	Ministry of Health	Ministry Of Health	Head of the Mental Health Division	Yes	No
72.	Ministry of Health	Ministry Of Health	Head of the Pharmaceutical Division	No	No
73.	Ministry of Health	Ministry Of Health	Head of the Regulatory Division	No	No
74.	Ministry of Health	Ministry Of Health	Head of the Rehabilitation Department	Yes	No
75.	Ministry of Health	Ministry Of Health	Manager of planning, development and construction Health institutions	No	No
76.	Ministry of Health	Ministry Of Health	Ombudsman for Medical Professions	Yes	Yes
77.	Ministry of Health	Ministry Of Health	Senior Deputy Director General , head of the Strategic and Economic Planning Administration	No	No
78.	Ministry of Health	Ministry Of Health	Senior Deputy Director General of Quality and Safety	Yes	No
79.	Sick Fund	Clalit Health Services	director of Eilat district	Yes	No
80.	Sick Fund	Clalit Health Services	director of Jerusalem district	No	No
81.	Sick Fund	Clalit Health Services	director of southern district	No	No
82.	Sick Fund	Clalit Health Services	director of Tel Aviv district	Yes	No
83.	Sick Fund	Clalit Health Services	director of the Dan Petah Tikva district	Yes	Yes

No.	Organization type	Organization	Position	Physician?	Specialist In Medical Administration?
84.	Sick Fund	Clalit Health Services	director of the central district	No	No
85.	Sick Fund	Clalit Health Services	director of the Sharon Samaria district	Yes	No
86.	Sick Fund	Clalit Health Services	CEO	No	No
87.	Sick Fund	Clalit Health Services	CFO	No	No
88.	Sick Fund	Clalit Health Services	Chief HR Officer	No	No
89.	Sick Fund	Clalit Health Services	Chief Innovation Officer & Deputy-DG	Yes	Yes
90.	Sick Fund	Clalit Health Services	Director of northern branch	Yes	No
91.	Sick Fund	Clalit Health Services	director of the Haifa and Western Galilee district	No	No
92.	Sick Fund	Clalit Health Services	Head of Hospitals Division	Yes	Yes
93.	Sick Fund	Clalit Health Services	head of marketing and patient experience division	No	No
94.	Sick Fund	Clalit Health Services	Head Of Procurment Division	No	No
95.	Sick Fund	Clalit Health Services	vp Digital & Technology	No	No
96.	Sick Fund	Leumit Health Services	Central District Manager	No	No
97.	Sick Fund	Leumit Health Services	CEO	No	No
98.	Sick Fund	Leumit Health Services	CFO	No	No
99.	Sick Fund	Leumit Health Services	Chief Medical Officer	Yes	No
100.	Sick Fund	Leumit Health Services	deputy director general and head of operation	No	No

No.	Organization type	Organization	Position	Physician?	Specialist In Medical Administration?
101.	Sick Fund	Leumit Health Services	head of the Jerusalem district	No	No
102.	Sick Fund	Leumit Health Services	North District Manager	No	No
103.	Sick Fund	Leumit Health Services	Southern District Manager	No	No
104.	Sick Fund	Maccabi Health Services	CEO	No	No
105.	Sick Fund	Maccabi Health Services	Chief Communications & Government Affairs Officer	No	No
106.	Sick Fund	Maccabi Health Services	Deputy CEO & head of health division	Yes	No
107.	Sick Fund	Maccabi Health Services	Deputy ceo & head of operation division	No	No
108.	Sick Fund	Maccabi Health Services	Deputy CEO, head of customer care and marketing division	No	No
109.	Sick Fund	Maccabi Health Services	Director, Finance and Hospitals Division	No	No
110.	Sick Fund	Maccabi Health Services	Head of the Central District	No	No
111.	Sick Fund	Maccabi Health Services	Head of the Jerusalem and Shfela District	No	No
112.	Sick Fund	Maccabi Health Services	Head of the North District	No	No
113.	Sick Fund	Maccabi Health Services	Head of the Sharon district	No	No
114.	Sick Fund	Maccabi Health Services	Head of the South District	No	No
115.	Sick Fund	Maccabi Health Services	VP and Head of the Technology Division	No	No
116.	Sick Fund	Maccabi Health Services	VP of Human Resources	No	No
117.	Sick Fund	Maccabi Health Services	VP of Innovation and Future Medicine	Yes	No

No.	Organization type	Organization	Position	Physician?	Specialist In Medical Administration?
118.	Sick Fund	Maccabi Health Services	VP of Strategy	No	No
119.	Sick Fund	Meuhedet Health Services	Central District Manager	No	No
120.	Sick Fund	Meuhedet Health Services	CEO	No	No
121.	Sick Fund	Meuhedet Health Services	Jerusalem District Manager	No	No
122.	Sick Fund	Meuhedet Health Services	Northern District Manager	No	No
123.	Sick Fund	Meuhedet Health Services	Southern District Manager	No	No
124.	Sick Fund	Meuhedet Health Services	VP, Director of the Customer Relations Division	No	No
125.	Sick Fund	Meuhedet Health Services	VP, Director of the Finance Division	No	No
126.	Sick Fund	Meuhedet Health Services	VP, Director of the Human Resources Division	No	No
127.	Sick Fund	Meuhedet Health Services	VP, Director of the Medical Division	Yes	Yes
128.	Sick Fund	Meuhedet Health Services	VP, Director of the Operations Division	No	No
129.	Sick Fund	Meuhedet Health Services	VP, Institutional Assets Director	No	No
